# Insanity and Inoculation

**Published:** 1894-12-22

**Authors:** 


### INSANITY AND INOCULATION.

An interesting discussion is at present occupying the attention of French specialists on insanity, certain savants claiming that insanity can be induced by inoculation. Heredity and organic disease of the nerves and nerve cells have long been well recognised causes of insanity, but insanity by inoculation is a somewhat novel departure in the ætiology of the condition. In certain diseases, such as typhoid, puerperal, and enteric fevers, poisons are produced in the body, which, as in the production of the analagous condition of delirium, can so destroy the nervous functions as to cause all the symptoms of insanity. From experiments on animals, it is claimed that insanity could probably be induced in a second person by injection into his veins of blood abstracted from a lunatic suffering from "auto-intoxication."

